# Broadband wave plates made by plasmonic metamaterials

**DOI:** 10.1038/s41598-018-19611-8

**Published:** 2018-01-18

**Authors:** Lin Chen, Xianmin Ke, Huijie Guo, Junhao Li, Xun Li, Lei Zhou

**Affiliations:** 1 Wuhan National Laboratory for Optoelectronics, Huazhong University of Science and Technology, Wuhan, 430074 China; 2 State Key Laboratory of Surface Physics and Key Laboratory of Micro and Nano Photonic Structures (Ministry of Education), Fudan University, Shanghai, 200433 China

## Abstract

Although metamaterials wave-plates have been demonstrated previously, many of them suffer from the issue of narrow bandwidth since they typically rely on resonance principles and thus exhibit inevitable frequency dispersions. Here, we show that the dispersion of spoof surface plasmon (SSP) mode supported by a fishbone structure can be freely modulated by varying the structural parameters. This motivates us to establish a general strategy of building broadband wave-plates by cascading two fishbone structures with different propagation constants of SSP modes. We derive a criterion under which the cross-polarization phase-difference across the whole device can maintain at a nearly constant value over a wide frequency band, with frequency dispersions in the two fishbone structures cancelled out. As an illustration, we design and fabricate an efficient microwave quarter-wave plate and experimentally characterize its excellent polarization-control performances over a broad frequency band (7–9.2 GHz). Our findings can stimulate making dispersion-controlled high-performance optical functional devices in different frequency domains.

## Introduction

The ability to manipulate the polarization states of electromagnetic (EM) waves is of central importance in both fundamental optics physics and photonics applications^1^. Compared with linear polarization, circular polarization is particularly important in sensing biological structures with chiral geometry^2^ and chiral imaging^3^. Conventionally, conversions between different types of polarizations are realized with wave plates made by birefringent crystals with cross-polarization phase-difference determined by the thickness and birefringence of the crystals. Unfortunately, such devices inherently exhibit narrow working bandwidth since the birefringent index is highly frequency-dependent.

Metamaterials (MTMs), artificial materials constructed by subwavelength-sized building blocks, have attracted intensive interests recently due to their strong abilities to control EM waves^4–7^. In particular, many MTM-based wave plates were proposed and/or demonstrated, which exhibit many advantages over conventional devices in terms of compactness, flexibility, and easy integration^8–24^. However, since the building blocks of MTMs are typically some resonant structures, such devices again exhibit limitted working bandwidths, since the inevitable frequency dispersions of MTMs make the cross-polarization phase-difference deviate quickly from the desired values at frequencies out of the designed working frequency. While plenty of works have been proposed to enlarge the working bandwidth of such MTM-based devices by cascading multiple resonant modes, many of them are for reflection geometry where the reflection amplitude is typically near 100% and one only need to control the dispersion of reflection phase^10–15^. For transmission geometry which is more useful in applications, one can not directly use this scheme since the transmission phase is locked with the amplitude and their frequency dispersions should be considered simultaneously^22–29^.

In this paper, we establish a general strategy to build high-performance broadband wave-plates based on spoof surface plasmon (SSP) modes with phase dispersions well controlled. Previous study has demonstrated that a single fishbone structure array supporting SSP modes fails to construct a broadband wave plate since the cross-polarization phase difference could not be kept constant within a wide spectral band^30,31^. We first show that the dispersions of SSP modes can be strongly modulated by the fishbone structural parameters^30–35^. Jointing two fishbone structures exhibiting opposite signs of frequency dispersions to form a single device, we show that the physical responses of such a device (e.g., cross-polarization phase difference and transmittance ratio) can be nearly dispersionless over a broad frequency range if the structural parameters of two fishbone structures satisfy certain conditions, thanks to the dispersion cancellation effect. As a proof-of-concept demonstration, we design and fabricate a microwave quarter-wave plate and experimentally show that it exhibits excellent linear-to-circular polarization conversion abilities over a wide frequency range (7–9.2 GHz). Our results, based on a general dispersion-control strategy, can inspire making broadband transmission-mode optical devices with other functionalities, such as half-wave plate, and in different frequency domains.

## Working Principle

**Figure 1:**
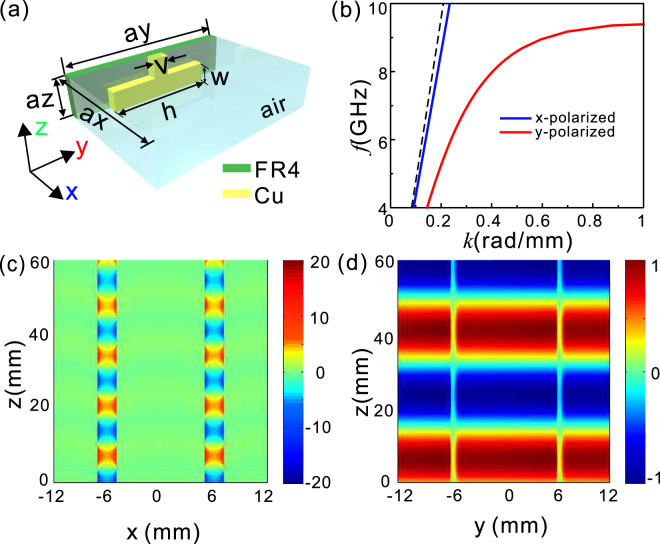
Dispersion curves and electric field distributions of x- and y- polarizations for the fishbone-shaped metallic structures. (**a**) Schematic of a unit cell of the fishbone-shaped metallic structures on a 0.6 mm-thick FR4 substrate. (**b**) The dispersion relationship of the array for x- (blue solid line) and y-polarization (red solid line). The black dashed line stands for the light line in the vacuum. The lattice constants along x, y, and z directions, ax, ay, and az, are set at 12, 12, and 0.5 mm, respectively. The geometrical parameters for the fishbone-shaped metallic structures, h, w, and v, are given as 9.9, 0.25, and 0.3 mm, respectively. The metallic structure is 17.5 μm-thick along x direction, ultra-thin with respect to the wavelength in free space. The dispersion curves are numerically solved by the Eigenmode Solver of CST MICROWAVE STUDIO. (**c**) Distribution of the real part of Ey in y-z plane at 8.5 GHz with y-polarized incidence. (**d**) Distribution of the real part of E_x_ in x-z plane at 8.5 GHz with x-polarized incidence.

For light incident upon birefringent metamaterials, it will encounter different effective refraction index coefficients for two orthogonal polarizations. Here we take arrays of fishbone structures supporting SSP modes as an example to illustrate how to manipulate the phase dispersion to function as a broadband quarter-wave plate. The unit cell of a fishbone structure is schematically shown in Fig. 1(a). It has been intensively demonstrated that, such a fishbone structure (not array) supports the propagation of high-confinement SSP mode at terahertz and microwave frequencies upon y-polarized incidence^33,34^. In our case, copper and FR4 are taken as the metallic layer and dielectric substrate with the conductivity and the relative permittivity of 5 × 10^7^ (Ωm)^−1^, 4.3 + 0.025i, respectively. To periodically arrange the fishbone structures in an array shown in Fig. 1(a), the SSP mode could be achieved by using a smaller gap separation to enhance the coupling between the symmetrical SSP modes. In contrast to y-polarized EM waves, the x-polarized EM waves pass through the array without exciting SSPs, and hence the dispersion curve is close to the light line. The simulation results in Fig. 1(b) demonstrate that significantly different propagation constants are produced, depending on whether the incident electric field is along x or y direction. The dispersion curve for y-polarization deviates significantly from the light line, while that for x-polarization nearly coincides with the light line. Figure 1(c) clearly indicates that the electric field intensity for y-polarized incidence is notably enhanced inside the gap between the adjacent fishbone structures. Meanwhile, the electric field distribution for x-polarized incidence is quite similar to that of the EM waves passing through the free space [Fig. 1(d)]. The prominent difference of the field intensity distributions for x- and y-polarized incidences in Fig. 1(c,d) confirms the birefringent nature of the array of fishbone-shaped metallic structures.

When the array of the fishbone-shaped metallic structures is illuminated with y-polarized incidence, the propagation constant along y direction, ${k}_{z}^{(y)}(f)$, is a highly nonlinear function of *f* and sensitively depends on the metallic structure generating SSPs, as well the lattice constant along y direction that controls the coupling between the SSP modes in the adjacent units. At frequencies a bit far away from the cut-off frequency of the SSPs, one can linearize ${k}_{z}^{(y)}(f)$ as

1 $${k}_{z}^{(y)}(f)\approx {k}_{z}^{(y)}({f}_{0})+(f-{f}_{0})2\pi {n}_{y}/c$$

where *f*_0_ is the reference frequency around *f*, *c* is the velocity of light in the air, and 2*πn*_*y*_/*c* is the slope of ${k}_{z}^{(y)}(f)$ with respect to *f*. As for x-polarized incidence, the EM waves will be transmitted with little influence from the metallic structure, hence the propagation constant, ${k}_{z}^{(x)}(f)$, is nearly a linear function of *f* and can be expressed as

2 $${k}_{z}^{(x)}(f)=2\pi {n}_{x}f/c$$

where *n*_*x*_ denotes the effective refraction index for x-polarized incidence.

Assuming that the array of fishbone-shaped metallic structures has a finite height of H along z direction, the phase difference between the two orthogonal polarizations for the transmitted light can be given as

3 $${\rm{\Phi }}=({k}_{z}^{(y)}-{k}_{z}^{(x)})H\approx [{k}_{z}^{(y)}({f}_{0})-2\pi {n}_{y}{f}_{0}/c+2\pi ({n}_{y}-{n}_{x})f/c]H$$

Considering the practical situations indicated in Fig. 1(b) that n_*y*_ is always larger than n_*x*_, Φ is increased monotonously within the considered frequency range. It can be easily inferred that, an array of fishbone-shaped metallic structures fails to generate flat phase difference between the two orthogonal polarizations due to the frequency dispersion, represented by the third term of Eq. (3).

**Figure 2:**
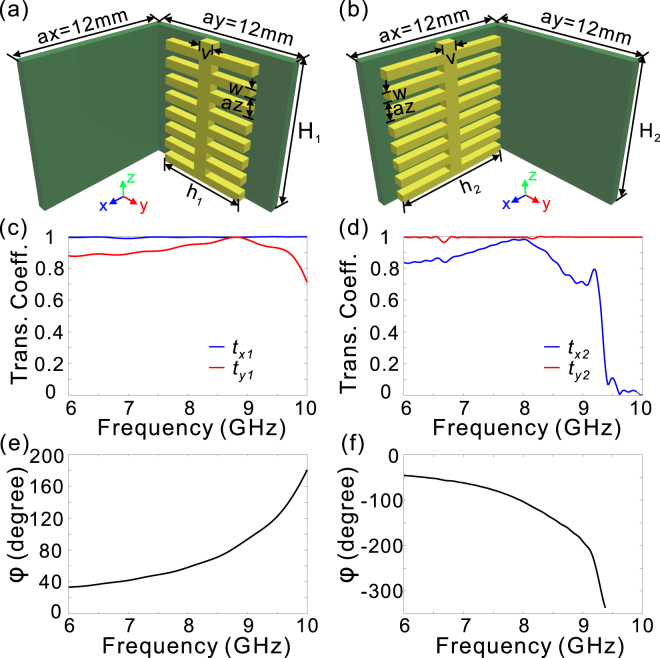
Transmission coefficients and phase differences of two orthogonal fishbone-shaped metallic arrays. (**a**,**b**) The unit cell of two arrays of fishbone-shaped metallic structures on the 0.6 mm-thick FR4 substrate, which are placed orthogonally and have the height of *H*_1_ (**a**) and *H*_2_ (**b**), respectively. (**c**,**d**) The transmission coefficients of *E*_*x*_ (*t*_*x*1_), and *E*_*y*_ (*t*_*y*1_) in (**a**) and (**b**), respectively. (**e**,**f**) The phase difference in (**a**) and (**b**), respectively. The geometrical parameters for the two arrays are given as *h*_1_ = 9.8 *mm*, *H*_1_ = 7.5 *mm* for (**a**), and *h*_2_ = 9.9 *mm*, *H*_2_ = 7.5 *mm* for (**b**), while the other structural parameters are the same as those in Fig. 1.

**Figure 3:**
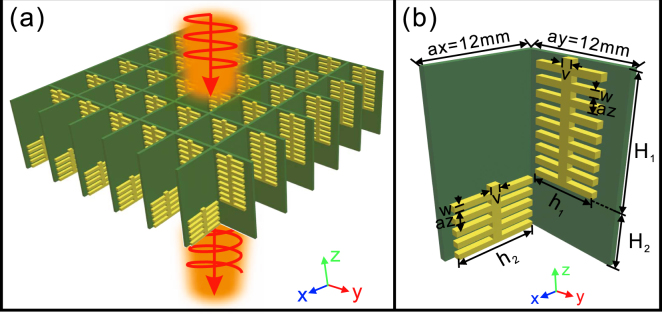
The schematics of the double-stacked arrays of fishbone-shaped metallic structures. (**a**,**b**) The double-stacked arrays of fishbone-shaped metallic structures (**a**) and the unit cell (**b**). The structural parameters are schematically shown in (**b**).

We then consider the transmission characteristics for two sets of fishbone-shaped metallic structure arrays placed orthogonally as shown in Fig. 2(a,b). It can be seen that high transmission efficiency can be maintained for both Ex and Ey components if the working frequency is away from the cut-off frequency [Fig. 2(c,d)], due to the low reflection at the incidence plane, and low transmission loss. On the basis of Eq. (3), the phase difference for the two arrays can be, respectively, derived as

4 $$\begin{array}{c}{{\rm{\Phi }}}_{{\rm{1}}}=[{k}_{z}^{(y)}({f}_{0})-2\pi {n}_{y1}{f}_{0}/c+2\pi ({n}_{y1}-{n}_{x1})f/c]{H}_{1}={k}_{1}{H}_{1}+2\pi {H}_{1}{\rm{\Delta }}{n}_{1}f/c\\ {{\rm{\Phi }}}_{{\rm{2}}}=[-{k}_{z}^{(x)}({f}_{0})+2\pi {n}_{x2}{f}_{0}/c-2\pi ({n}_{x2}-{n}_{y2})f/c]{H}_{2}=-{k}_{2}{H}_{2}-2\pi {H}_{2}{\rm{\Delta }}{n}_{2}f/c\end{array}$$

where ${k}_{1}={k}_{z}^{(y)}({f}_{0})-2\pi {n}_{y1}{f}_{0}/c$, Δ*n*_1_ = *n*_*y*1_ − *n*_*x*1_, ${k}_{2}={k}_{z}^{(x)}({f}_{0})-2\pi {n}_{x2}\,{f}_{0}/c$, and Δ*n*_2_ = *n*_*x*2_ − *n*_*y*2_. For both cases the phase differences are monotonously changed within the considered frequency range, and their signs are opposite since the two arrays are placed orthogonally [Fig. 2(e,f)]. Therefore, when we connect two such arrays with the height of *H*_1_ and *H*_2_ [Fig. 3], and neglect the reflections at the interface between the two arrays, the total phase difference accumulation is given by

5 $${{\rm{\Phi }}}_{tot}=({k}_{1}{H}_{1}-{k}_{2}{H}_{2})+2\pi f/c({\rm{\Delta }}{n}_{1}{H}_{1}-{\rm{\Delta }}{n}_{2}{H}_{2})$$

It is interesting to note that Δ*n*_1_ and Δ*n*_2_ are both positive values within the considered frequency range [Fig. 2(c,d)], indicating that the phase difference dispersion can be more or less cancelled out. To achieve a flat phase accumulation within the considered frequency range, one must eliminate the frequency dispersion, yielding the first condition

6 $${\rm{\Delta }}{n}_{1}{H}_{1}-{\rm{\Delta }}{n}_{2}{H}_{2}=0$$

Meanwhile, the absolute height of each array is determined by the desired functionality,

7 $${{\rm{\Phi }}}_{tot}={k}_{1}{H}_{1}-{k}_{2}{H}_{2}$$

with Φ_*tot*_ = *π*/2 (or *π*), if a quarter-wave (or half-wave) plate is desired.

## Results and Discussion

### Simulation Results

**Figure 4:**
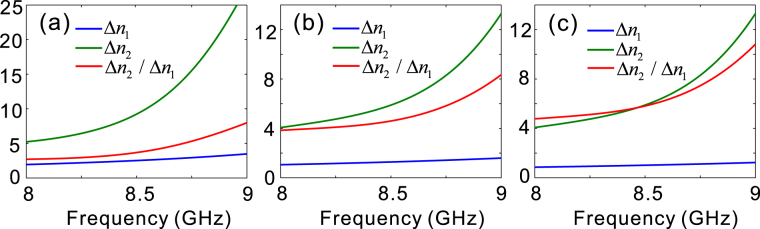
Δn_1_, Δn_2_, and Δn_2_/Δn_1_ of three pairs of arrays of fishbone-shaped metallic structures. Δ*n*_1_, Δ*n*_2_, and Δ*n*_2_/Δ*n*_1_ as a function of frequency for (**a**) *h*_1_ = 8.6 mm, and *h*_2_ = 9.9 mm; (**b**) *h*_1_ = 7.6 mm, and *h*_2_ = 9.6 mm; (**c**) *h*_1_ = 7.2 mm, and *h*_2_ = 9.6 mm. The other geometrical parameters are the same as those in Fig. 1. Δ*n*_2_/Δ*n*_1_ in (**a**), (**b**), and (**c**) is approximately 4, 5, and 6 at 8.55 GHz.

In addition to the requirement of constant phase difference of π/2, a broadband quarter-wave plate should keep the transmission ratio between the two orthogonal polarizations fixed over a wide spectral band. We know from Fig. 2(c,d) that, these transmission conditions can be basically satisfied below the cut-off frequency (9 GHz). Δ*n*_1_, Δ*n*_2_ and Δ*n*_2_/Δ*n*_1_ as a function of *f* are depicted in Fig. 4(a). Choosing the reference frequency as *f*_0_ = 8.55 GHz, Δ*n*_2_/Δ*n*_1_ is about 4. As a result, *H*_1_ and *H*_2_ are estimated to be 26 and 6.5 mm, according to Eqs (6, 7). Varying the structural parameters of *h*_1_ and *h*_2_, for each array, we are able to achieve different values of Δ*n*_2_/Δ*n*_1_ around 8.55 GHz [Fig. 4(b,c)]. In the same way, we can get the values of *H*_1_ and *H*_2_ that enable constant phase delay of π/2 around 8.55 GHz. In a word, it is highly expected to develop a broadband quarter-wave plate with the double-stacked arrays of fishbone-shaped metallic structures in the considered frequency range.

**Figure 5:**
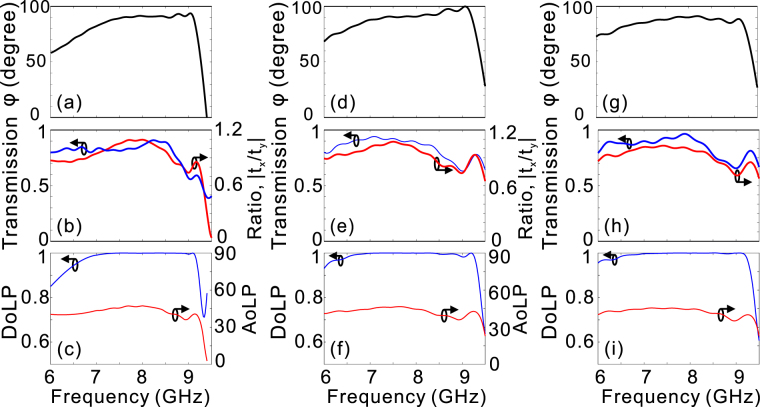
The simulated performance for the three designed quarter-wave plates. The phase difference (**a**,**d**,**g**), and transmittance, amplitude ratio t_x_/t_y_ (**b**,**e**,**h**), versus the light frequency. DoLP and AoLP (**c**,**f**,**i**) as a function of the light frequency with left-circularly polarized waves normally incident from -z direction. The heights for each array of fishbone-shaped metallic structures are H_1_ = 26 mm and H_2_ = 6.5 mm (**a**–**c**), H_1_ = 43 mm and H_2_ = 8.5 mm (**d**–**f**), and H_1_ = 48.5 mm and H_2_ = 8 mm (**g**–**i**), respectively.

By using above-mentioned three sets of geometrical parameters to form the double-stacked arrays of fishbone-shaped metallic structures, the simulated phase delay exhibits flat response within the design frequency range around 8.55 GHz [Fig. 5(a,d,g)]. If the phase difference for a quarter-wave plate is defined as 90° ± 10°, the retrieved operation bandwidths are 6.9–9.2 GHz, 6.5–9.3 GHz, and 6.5–9.2 GHz, respectively, which are up to 28.6%, 35.4%, and 34.4% of the central frequencies. The operation bandwidths can be kept as high as 23.3% (7.2–9.1 GHz), 22.2% (6.8–8.5 GHz), and 27.2% (7.0–9.2 GHz) of the central frequency, even if the phase delay for a quarter-wave plate is more strictly defined as 90° ± 5°. Besides, the corresponding transmittance exceeds 0.8 in the design frequency range [Fig. 5(b,e,h)]. The fact that the double-stacked structures suffer from lower transmittance at higher frequencies can be attributed to the larger absorption loss when the light frequency is near the cut-off frequencies of the SSP mode. Figure 5(b,e,h) also present that the amplitude ratio between Ex and Ey is close to one within the frequency range of interest. In a word, the double-stacked arrays satisfy all the prerequisites to construct a good quarter-wave plate.

**Figure 6:**
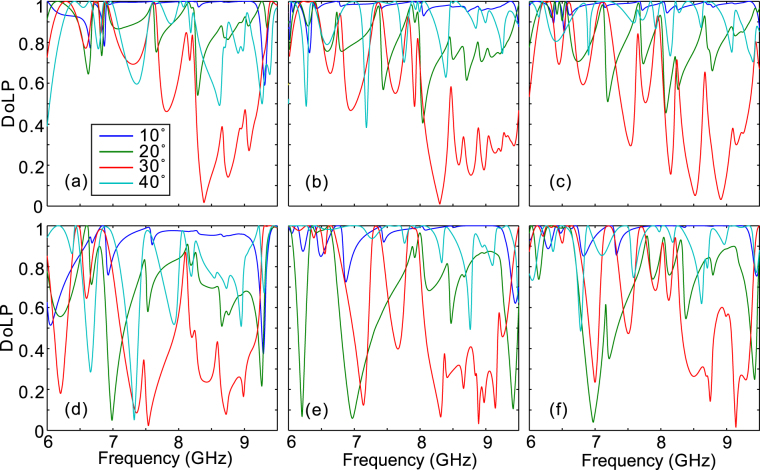
The simulated device performance under oblique incidences. DoLP as a function of light frequency for the first (**a**,**d**), second (**b**,**e**), and third (**c**,**f**) cases as the oblique angle is varied in x-z (**a**–**c**) and y-z (**d**–**f**) planes.

To quantitatively evaluate the performance of the designed quarter-wave plate based on the double-stacked arrays of fishbone-shaped metallic structures, we have calculated the degree of linear polarization (DoLP) and the angle of linear polarization (AoLP) with left-circularly polarized light wave incident from -z direction. DoLP is used to evaluate the degree of linear polarization of the transmitted wave, while AoLP represents the polarization angle of the linearly polarized light relative to x-axis. DoLP and AoLP are defined as $DoLP=\sqrt{{s}_{1}^{2}+{s}_{2}^{2}}/{s}_{0}$ and $AoLP=0.5{\tan }^{-1}({s}_{2}/{s}_{1})$, respectively, where *s*_0_, *s*_1_, and *s*_2_ are the Stokes parameters given by *s*_0_ = |*E*_*x*_|^2^ + |*E*_*y*_|^2^, *s*_1_ = |*E*_*x*_|^2^ − |*E*_*y*_|^2^, and ${s}_{2}={E}_{x}{E}_{y}^{\ast }+{E}_{x}^{\ast }{E}_{y}$^36^. Here, ${E}_{x}^{\ast }$ and ${E}_{y}^{\ast }$ denote the complex conjugate of *E*_*x*_ and *E*_*y*_, respectively. It can be observed from Fig. 5(c,f,i) that the bandwidth over which the DoLP is nearly unity (>0.99) is consistent with π/2 phase bandwidth, indicating the output EM wave is almost linearly polarized. Furthermore, the AoLP is approximately 45° accordingly [see Fig. 5(c,f,i)], originating from the nearly equal transmission coefficients of Ex and Ey [Fig. 5(b,e,h)]. The weak dependence of AoLP on light frequency enables fixing the fast and slow axes of a quarter-wave plate, which is beneficial for practical applications. We have noted recent studies on quarter-wave plates with plasmonic metasurfaces have been recently proved incapable of keeping the AoLP stable within the phase bandwidth of operation due to the strong dispersion in the transmission/reflection coefficients^14,23–25,37^. While the proposed double-stacked array above mentioned operates well under normal incidence, the device performance shows rather sensitiveness to the oblique angle. It can be seen from Fig. 6 that, the DoLP deviates significantly from one for most of the frequency of interest even if the incident angle is slightly shifted to 20°.

### Experimental Results

**Figure 7:**
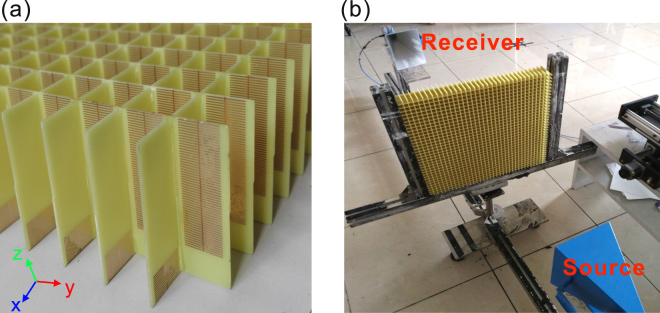
The first sample and the measurement setup. (**a**) The schematic of the fabricated quarter-wave plate for the first sample, (**b**) the experimental measurement setup.

**Figure 8:**
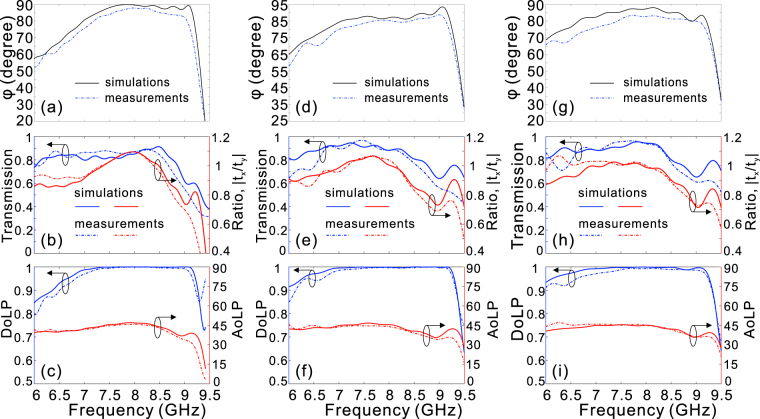
Comparison between the numerical simulations and experimental measurements. The phase differences (**a**,**d**,**g**), transmission and ratio |*t*_*x*_/*t*_*y*_| (**b**,**e**,**h**), and DoLP and AoLP (**c**,**f**,**i**) for the first (**a**–**c**), the second (**d**–**f**), and the third (**g**–**i**) samples. In the simulations, the actual geometrical parameters are taken into account.

Choosing the designed geometrical parameters as mentioned above, we have successfully designed and fabricated 3 samples to realize broadband linear-to-circular polarization conversion around 8 GHz. The samples with the size 420 × 420 mm^2^ were fabricated with printed circuit board (PCB) technology, where the first sample is depicted in Fig. 7(a). The actual thickness of the FR4 board is 0.56 mm, 0.04 mm thinner than the designed value. Due to the fabrication error of the PCB technology, the heights for the three fabricated samples are 32.3 mm, 51.3 mm, and 56.3 mm, respectively. The experimental measurement setup is illustrated in Fig. 7(b). The fabricated device is vertically fixed between two standard gain horn antennas, of which one is the source and the other is the receiver. The measured phase bandwidths (defined as 90° ± 10°) for the three samples are 7.2–9.1 GHz, 7–9.2 GHz, and 7.4–9 GHz, respectively [Fig. 8(a,d,g)]. Meanwhile, the measured transmission is basically larger than 0.8 for most of frequencies below 9 GHz and the amplitude ratio between Ex and Ey is slightly oscillated around one within the design frequency range [Fig. 8(b,e,f)]. More importantly, highly linearly polarized EM waves are output when the fabricated samples are illuminated upon the circularly polarized EM waves, as the DoLP is nearly kept at one (>0.98 in the experiment) within the phase bandwidth [Fig. 8(c,f,i)]. Further, the measured AoLP presents weak dependence on light frequency, which is highly expected to construct an effective quarter-wave plate for practical applications. In all the cases, the measured device performances are basically consistent with those from numerical simulations by taking into account the actual structural parameters. The deviation of measured and designed device performance may come from the fabrication errors and measurement precision. It should be emphasized here, the transmission efficiency for each case can be kept at a high level (>80% for most of the operation frequencies) since the considered frequency is far from the cut-off frequency.

## Conclusion

We proposed a general strategy for designing transmission-mode broadband wave-plates by integrating two fishbone structures with sides walls decorated with different plasmonic metamaterials. As the geometrical parameters of those plasmonic decorations satisfy certain conditions, we find that frequency dispersions contributed by two fishbone structures can exactly cancel out, leading to a nearly dispersionless value of cross-polarization phase-difference across the whole device within a broad frequency range. As a demonstration of our general strategy, we designed and fabricated a quarter-wave plate in the microwave regime, and experimentally demonstrated its excellent polarization conversion capabilities within a broad frequency band (7–9.2 GHz). We have noted recent studies show that the reflection at the interface between air and the fishbone structure arrays can be significantly reduced by using gradient fishbone structures^31,35^, indicating the promising way to further enhance the transmission for the present configuration. It is also worth noting here broadband quarter-wave plates have been proposed by integrating two metal wire-grid structures that have opposite signs of cross-polarization phase-difference so that the total phase dispersion is cancelled out^19^. However, the required phase delay for a quarter-wave plate is achieved with the sacrifice of the transmission amplitude by increasing the filling ratio of metal stripes. As a result, the reported conversion efficiency (~50% within the operation frequency range) is significantly lower than the present case. Finally, we emphasize that our proposal can also be applied to design other EM components with broadband features, and be scaled down to high frequencies as well. For the future experimental implementation of such proposal at optical frequencies, the fishbone structures might be fabricated with the direct laser writing followed by electrochemical deposition of metal^7^.

## Methods

All samples were fabricated using 0.56-mm-thick FR4 films with one side covered by 17-µm-thick copper films, with printed circuit board (PCB) technology. In the measurements, the incident EM waves were generated by a horn antenna placed 2 m away from the samples, and the transmission coefficients were measured with another identical horn antenna, which was placed at the same distance but the opposite direction to the samples. Both antennas were connected to a vector network analyzer (Agilent E8362C PNA). The received signals were normalized against references measured when the samples were removed.
